# Synthesis, Characterization, and Performance Evaluation of Sulfur-Containing Diphenylamines Based on Intramolecular Synergism

**DOI:** 10.3390/molecules23020401

**Published:** 2018-02-13

**Authors:** Jun-Bo He, Hao Shi, Yue Wang, Xin-Lei Gao

**Affiliations:** 1 Key Laboratory for Deep Processing of Major Grain and Oil, Ministry of Education, College of Food Science & Engineering, Wuhan Polytechnic University, Wuhan 430023, China; junb112he@whpu.edu.cn (J.-B.H.); go_live@yeah.net (H.S.); 2 Department of Chemical and Environmental Engineering, Wuhan Polytechnic University, Wuhan 430023, China; yuewang27@163.com

**Keywords:** sulfur-containing 4-aminodiphenylamine, dual functional antioxidants, antioxidant mechanism, intramolecular synergism

## Abstract

To obtain novel structural antioxidants that have different antioxidant mechanisms, four 2-(alkylthio)-*N*-(4-(phenylamino)phenyl)acetamides **2a**–**d** as dual functional antioxidants are designed, synthesized, and confirmed by ^1^H-NMR, FTIR, MS, and elemental analysis. The antioxidant behavior of compounds **2a**–**d** as additives of base oil triisodecyl trimellitate (TIDTM) is evaluated by non-isothermal and isothermal DSC analyses. The results showed all compounds can greatly increase the incipient oxidation temperature (IOT) and oxidation induction time (OIT) of TIDTM, especially, compound **2c** exhibited an OIT value of 72.5 min at 230 °C, which is almost 28 times the length of TIDTM. Moreover, compounds **2a**–**d** do not affect the tribological performance of TIDTM. The mechanism of antioxidants involved an intramolecular synergism are proposed. This work demonstrates compound **2c** can be used as a novel potential antioxidant additive of TIDTM; in addition, it would inspire the emergence of highly potent antioxidants with different antioxidant mechanisms.

## 1. Introduction

Synthetic ester oils, which have been widely used in the world, have many advantages over mineral base oils, such as excellent viscosity–temperature relationship, broad operating temperature range, and good low temperature fluidity [1,2,3]. However, synthetic ester base oils are usually sensitive to chemical oxidation reactions in the presence of oxygen, water, and metals under elevated temperature without the addition of antioxidants.

The most effective way to enhance the oxidation stability is introducing antioxidants into the formulations [4,5,6]. According to the oxidation mechanism of lubricants, antioxidants are classified into three types [7,8]: free radical scavengers, hydroperoxide decomposers, and metal deactivators. Among them, free radical scavengers and hydroperoxide decomposers are the most used antioxidants in lubricants. Representative free radical scavengers are hindered phenol derivatives [9,10] and arylamine derivatives [11,12,13,14], and the commonly used hydroperoxide decomposers are alkyl sulfides or alkyl polysulfides [7,15]. Thus far, most antioxidants are simple functional antioxidants which display the same action mechanism, thus their protection effectiveness is greatly affected. To resolve this problem, dual functional or more functional oxidation inhibitors based on synergism may greatly increase the antioxidant potency.

**Figure 1:**
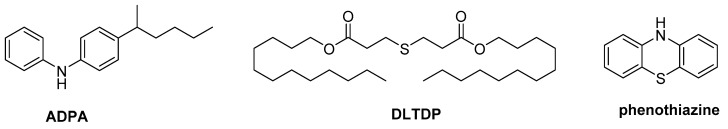
Some representative antioxidants.

Based on the abovementioned design concept of antioxidants, some dual functional antioxidants, such as compounds containing diphenylamines and hindered phenols simultaneously [16,17,18], have been synthesized and showed improved antioxidant properties. However, these antioxidants are homosynergism which displayed the same action mechanism, and the synergistic effectiveness is limited. Therefore, dual functional antioxidants with different action mechanism would display more effective antioxidant activity since they are heterosynergistic [19]. Two strategies can be used to achieve the purpose, one is mixing two kinds of antioxidant with different action mechanism, such as alkylated diphenylamines (**ADPA**) and dilauryl thiodipropionate (**DLTDP**) [20] (Figure 1), and the other one is integrating two different functional groups into one molecule to form intramolecular synergism. Phenothiazine, containing arylamine and sulfide groups, has been reported as dual functional oxidation inhibitor [21] (Figure 1). However, since the sulfide group is aromatic, the hydroperoxide decomposing action is limited because it lacks the β-hydrogen atoms, which means the β-elimination reaction cannot take place to form the further hydroperoxide decomposing sulfinic and sulfenic compounds [7,22]. Hence, to design and develop new structural sulfur-containing diphenylamines compounds, which may lead to novel compounds with higher antioxidant potency owing to the intramolecular synergism, is becoming an urgent project.

**Figure 2:**
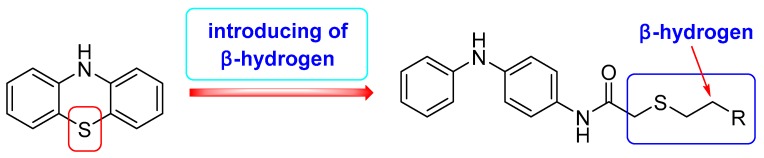
Design of the sulfur-containing 4-aminodiphenylamine derivatives.

Herein, we report, for the first time, the design and synthesis of 2-(alkylthio)-*N*-(4-(phenylamino)phenyl)acetamides, which simultaneously have the diphenylamino group and sulfur ether group and the antioxidant character. As dual functional oxidation inhibitors with different action mechanisms (Figure 2), the sulfur ether moiety, which has β-hydrogen atoms, can promote the hydroperoxide decomposing action by β-elimination reaction. Their tribological activity and antioxidant behavior in commercial synthetic ester base oil triisodecyl trimellitate (TIDTM) are investigated. Furthermore, the possible antioxidant mechanism is proposed.

## 2. Results and Discussion

### 2.1. Chemistry

**Scheme 1:**
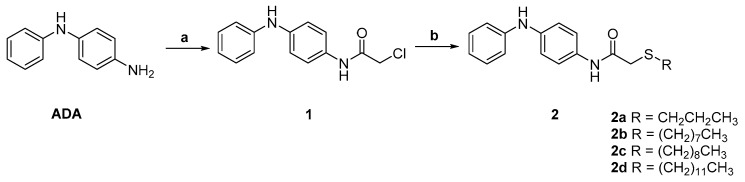
Regents and conditions: (**a**) chloroacetyl chloride, Et_3_N, CH_2_Cl_2_, 0 °C; and (**b**) R–SH, NaOH, C_2_H_5_OH, reflux.

Generally, to gain better solubility and higher thermal stability, diphenylamine antioxidants should be alkylated with certain length of hydrocarbon chain [16]. In this work, we introduce four alkyl hydrosulfides (C3, C8, C9, and C12) into the diphenylamine to study the influence of the length of carbon chain on the antioxidant behavior. The synthetic route employed to obtain compounds **2a**–**d** is depicted in Scheme 1. First, the intermediate 2-chloro-*N*-(4-(phenylamino)phenyl)acetamide (**1**) was synthesized from 4-aminodiphenylamine (**ADA**) with chloroacetyl chloride with triethylamine as base, and then reacted with four alkyl hydrosulfides in refluxing ethanol using NaOH as base to give compounds **2a**–**d**.

The structures of compounds **2a**–**d** were characterized by ^1^H-NMR, FTIR, MS, and elemental analysis. The protons shown in ^1^H-NMR spectra are exactly in accordance with the structures of compounds **2a**–**d**. The ^1^H-NMR spectra showed the presence of nitrogen protons (7.95–9.84 ppm), aromatic protons (6.74–7.47 ppm), and methylene protons (–COCH_2_–, 3.20–3.24 ppm). Moreover, the alkyl proton signals located at δ (0.84–2.61 ppm). For the FTIR spectra of compounds **2a**–**d**, the characteristic peaks at 3413–3270, 1654–1600, 1554–1546, and 1395–1315 cm^–1^ are related to the stretching vibration of N–H, C–N in CONH, C=C, and C–N, respectively. The C–H stretching of CH_2_ and CH_3_ are related to the peaks at 2915 and 2850 cm^–1^. The mass spectrum of all compounds showed molecular ion peak at *m/z* = [M + H], which is agree with the molecular formula.

### 2.2. Tribological Properties of Compounds ***2a**–**d***

**Figure 3:**
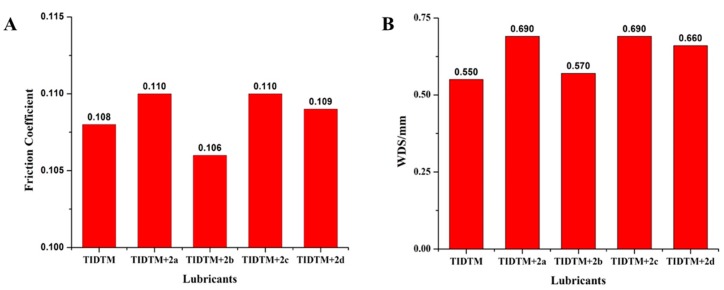
Friction coefficient (**A**); and wear scar diameter (**B**) under the lubrication of TIDTM and TIDTM containing 1 wt% **2a**–**d**, respectively.

As good antioxidant additive for lubricating oil should show good tribological properties or at least not be harmful to the tribological properties of the base oil. Therefore, the tribological properties of compounds **2a**–**d** in TIDTM were first studied and the results are shown in Figure 3a,b. As shown in Figure 3, the effects of compounds **2a**–**d** on the friction coefficient and wear scar diameter (WSD) have similar trend. Among the four compounds **2a**–**d**, compound **2b** with eight-carbon chain has the best tribological properties, which slightly decrease the friction coefficient of the base oil and have the lowest WSD value in the series. However, the three other compounds increase the friction coefficient and WSD of the base oil slightly.

### 2.3. Antioxidant Behavior of Compounds ***2a**–**d***

The antioxidant activity of compounds **2a–d** in TIDTM were further evaluated. ADA was used as reference antioxidant. The concentration of all additives in TIDTM base oil was 1 wt%. The incipient oxidation temperature (IOT) and oxidation induction time (OIT) were determined by DSC. A higher IOT and longer OIT mean better oxidation stability of the base oil.

**Figure 4:**
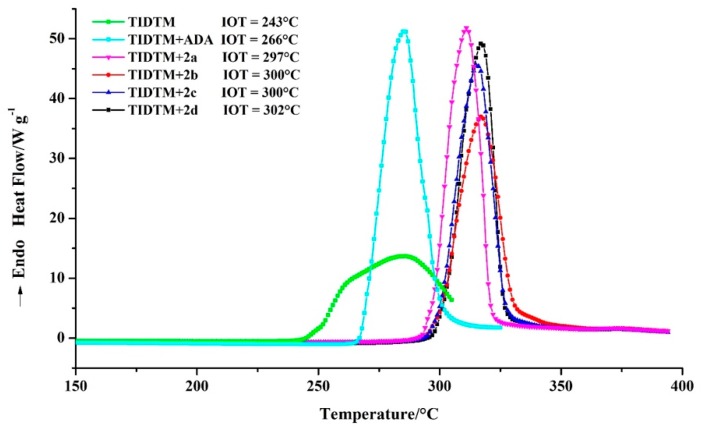
The incipient oxidation temperatures of TIDTM and TIDTM containing 1 wt% ADA and compounds **2a**–**d**, respectively.

First, the IOT performance of compounds **2a**–**d** and **ADA** in TIDTM is illustrated in Figure 4. The IOT value of base oil was only 243 °C, but the IOT values of base oil increased to 266 °C, 297 °C, 300 °C, 300 °C, and 302 °C along with the addition of **ADA** and compounds **2a**–**d**, respectively. The greatly increased IOT values of compounds **2a**–**d** compared to **ADA** indicate the introducing of alkyl hydrosulfides can remarkably increase the antioxidant potency. Moreover, the almost identical IOT values of compounds **2a**–**d** in TIDTM indicate that the length of carbon chain does not affect the antioxidant ability, which means the molecular weights of compounds **2a**–**d** have no correlation to the antioxidant potency.

**Figure 5:**
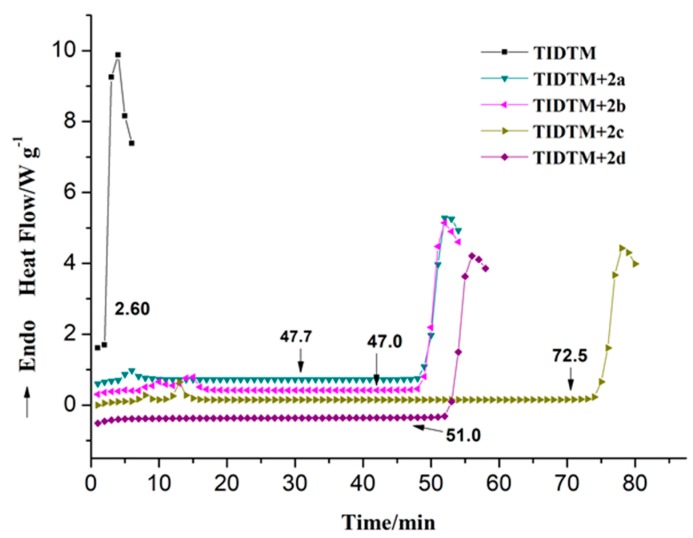
Oxidation induction time of TIDTM and TIDTM containing 1 wt% compounds **2a**–**d** at 230 °C, respectively.

The OIT values of compounds **2a**–**d** in TIDTM are shown in Figure 5. The base oil of TIDTM only has an OIT value of 2.6 min, however, the addition of compounds **2a**–**d** (1 wt% concentration) increased the OIT values of TIDTM to 47.67 min, 47 min, 72.5 min, and 51 min, respectively, which were 45.07 min, 44.4 min, 69.9 min, and 48.4 min longer than TIDTM without antioxidants. Especially, compound **2c** with the alkyl chain length of nine displayed the longest OIT value of 72.5 min, which is almost 28 times the length of TIDTM. These results illustrate that compound **2c** can effectively maintain the oxidant stability of TIDTM, and the design of integrating sulfur ether and diphenylamine as dual functional antioxidant is rational.

### 2.4. Antioxidant Mechanism of Compound ***2c***

**Scheme 2:**
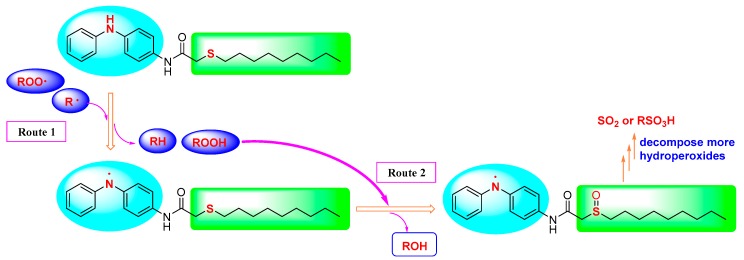
Possible mechanism of the dual functional antioxidant.

Based on the above investigations and previous reports [7,20], a mechanism of intramolecular synergistic effect is proposed, as shown in Scheme 2. As previously reported, for the oxidation mechanism of base oil [7,8], free radicals were generated primarily in the initiation stage, and then after hydroperoxides. Therefore, the diphenylamine moiety would act as the primary antioxidant (free radical scavenger) by donating hydrogen atom to form an aminyl radical, while the free radicals are terminated (Route 1 in Scheme 2). On the other hand, the hydroperoxides (ROOH), formed by alkyl peroxy radicals in the oxidation process, will be decomposed to more stable materials by the sulfur ether moiety of compound **2c** (Route 2 in Scheme 2) [7,20], along with the formation of sulfoxide intermediate. The subsequent reaction of sulfoxide intermediate is the intramolecular β-hydrogen elimination, leading to the formation of sulfenic acid (RSOH), which can be oxidized to sulfininc acid (RSO_2_H) and sulfonic acid (RSO_3_H) by the hydroperoxides. Besides, the sulfininc acid (RSO_2_H) may decompose to form sulfur dioxide (SO2) at elevated temperature which is a powerful hydroperoxide decomposer. Through these reactions, the oxidation process was significantly slowed or inhibited by the dual functional antioxidant compound **2c** through the intramolecular synergistic effect with different action mechanism.

The intramolecular synergism was validated by the IOT values of compounds **2a**–**d** in TIDTM, as depicted in Figure 4. When **ADA** was used as reference antioxidant, the IOT value of TIDTM can be increased for 23 °C because of its limited antioxidant activity. However, introducing alkyl sulfur ether can greatly enhance the antioxidant activity of compounds **2a**–**d** for their synergism, which makes the IOT values of TIDTM greatly extended.

Unlike reported intermolecular synergism for the physical mixture [20,23], the intramolecular dual functional antioxidants (compounds **2a**–**d**) with different action mechanism have special advantages. First, intramolecular synergism is more effective than intermolecular synergism, because the sulfur ether moiety can decompose the hydroperoxides immediately and thus avoid the intermolecular contact probability. Second, to achieve the same antioxidant activity, the mass of antioxidant that needs to be added is less, since they were bonded in one molecule. In brief, compounds **2a**–**d** have potent oxidative resistance capacity due to intramolecular synergism.

## 3. Materials and Methods

### 3.1. Materials

All reagents (purchased from Aladdin Chemical Corporation, Shanghai, China) used for preparing antioxidants were of analytical grade and used without further purification. Commercial synthetic ester base oil of TIDTM was used as the base oil without further treatment. ^1^H-NMR spectra were recorded in DMSO-*d*_6_ solution on a Varian Mercury-Plus 400 spectrometers (Varian, Palo Alto, CA, USA) at 400 MHz and chemical shifts were recorded in parts per million (ppm) with TMS as the internal reference. FTIR spectra were recorded on a Thermo Nicolet NEXUS 670 FTIR Raman spectrometer (Thermo Nicolet Corporation, Madison, WI, USA). Mass spectra (MS) were obtained on a Finnigan LTQ XL mass spectrometer (Palo Alto, CA, USA) and signals were given in *m/z*. Elemental analysis were performed with a Vario EL cube CHNOS elemental analyzer (Elementar, Hanau, Germany).

### 3.2. Preparation of 2-Chloro-N-(4-(phenylamino)phenyl)acetamide *(**1**)*

Chloroacetyl chloride (2.26 g, 0.02 mol) was added dropwise as a solution in CH_2_Cl_2_ (10 mL) to a solution of 4-aminodiphenylamine (3.68 g, 0.02 mol) and triethylamine (2.02 g, 0.02 mol) in CH_2_Cl_2_ (60 mL) at 0 °C, and then the mixture was stirred for 2 h. After that, the mixture was concentrated, poured into cold water (50 mL), and the precipitate was collected by filtration and dried. Recrystallization with ethanol afforded the desired compound **1** (4.21 g, 0.016 mol, 81%) as a gray solid, m.p. 150–152 °C (yield 82%, m.p. 144–145 °C, reported [24]).

### 3.3. General Procedure for the Preparation of Compounds ***2a**–**d***

A solution of compound **1** (0.78 g, 3 mmol), corresponding alkyl hydrosulfide (3 mmol) and NaOH (0.14 g, 3.6 mmol) in ethanol (30 mL), was heated under reflux until the reaction was complete based on TLC monitoring. Then, the mixture was cooled to room temperature, and the precipitate was collected by filtration and dried at vacuum condition. Compounds **2a**–**d** were collected by crystallization.

*N-(4-(Phenylamino)phenyl)-2-(propylthio)acetamide* (**2a**). Off-white solid, yield: 51%, m.p. 81–83 °C; ^1^H-NMR (DMSO-*d*_6_, 400 MHz): δ 9.85 (s, 1H), 8.00 (s, 1H), 7.43 (d, *J* = 8.0 Hz, 2H), 7.17 (t, *J* = 8.0 Hz, 2H), 6.99 (dd, *J* = 12.0, 8.0 Hz, 4H), 6.74 (t, *J* = 8.0 Hz, 1H), 3.24 (s, 2H), 2.60 (t, *J* = 8.0 Hz, 2H), 1.54–1.63 (m, 2H), 0.94 (t, *J* = 8.0 Hz, 3H). FTIR (KBr, cm^–1^) ν 3406, 3286, 2919, 2857, 1654, 1554, 1315. MS *m/z*: 301.14 [M + H]^+^. Anal. Calcd For C_17_H_20_N_2_OS: C, 67.97; H, 6.71; N, 9.32. Found: C, 67.72; H, 6.82; N, 9.13.

*2-(Octylthio)-N-(4-(phenylamino)phenyl)acetamide* (**2b**). Off-white solid, yield: 84%, m.p. 79–81 °C; ^1^H-NMR (DMSO-*d*_6_, 400 MHz): δ 9.84 (s, 1H), 8.00 (s, 1H), 7.43 (d, *J* = 8.0 Hz, 2H), 7.17 (t, *J* = 8.0 Hz, 2H), 6.99 (t, *J* = 8.0 Hz, 4H), 6.74 (t, *J* = 8.0 Hz, 1H), 3.24 (s, 2H), 2.61 (t, *J* = 8.0 Hz, 2H), 1.52–1.57 (m, 2H), 1.24–1.33 (m, 10H), 0.85 (t, *J* = 8.0 Hz, 3H). FTIR (KBr, cm^–1^) ν 3405, 3274, 2919, 2850, 1650, 1546, 1396. MS *m/z*: 371.28 [M + H]^+^. Anal. Calcd For C_22_H_30_N_2_OS: C, 71.31; H, 8.16; N, 7.56. Found: C, 71.15; H, 8.27; N, 7.46.

*2-(Nonylthio)-N-(4-(phenylamino)phenyl)acetamide* (**2c**). Off-white solid, yield: 89%, m.p. 88–90 °C; ^1^H-NMR (DMSO-*d*_6_, 400 MHz): δ 9.84 (s, 1H), 8.00 (s, 1H), 7.43 (d, *J* = 8.0 Hz, 2H), 7.17 (t, *J* = 8.0 Hz, 2H), 6.99 (t, *J* = 8.0 Hz, 4H), 6.74 (t, *J* = 8.0 Hz, 1H), 3.24 (s, 2H), 2.61 (t, *J* = 8.0 Hz, 2H), 1.54 (d, *J* = 8.0 Hz, 2H), 1.23–1.33 (m, 12H), 0.85 (t, *J* = 8.0 Hz, 3H). FTIR (KBr, cm^–1^) ν 3413, 3371, 3270, 2919, 2850, 1650, 1550, 1315. MS *m/z*: 385.19 [M + H]^+^. Anal. Calcd For C_23_H_32_N_2_OS: C, 71.83; H, 8.39; N, 7.28. Found: C, 71.65; H, 8.54; N, 7.43.

*2-(Dodecylthio)-N-(4-(phenylamino)phenyl)acetamide* (**2d**). Off-white solid, yield: 61%, m.p. 94–96 °C; ^1^H-NMR (DMSO-*d*_6_, 400 MHz): δ 9.83 (s, 1H), 7.99 (s, 1H), 7.43 (d, *J* = 8.0 Hz, 2H), 7.17 (t, *J* = 8.0 Hz, 2H), 6.99 (t, *J* = 8.0 Hz, 4H), 6.74 (t, *J* = 8.0 Hz, 1H), 3.23 (s, 2H), 2.61 (t, *J* = 8.0 Hz, 2H), 1.51–1.58 (m, 2H), 1.23–1.32 (m,18H), 0.84 (t, *J* = 8.0 Hz, 3H). FTIR (KBr, cm^–1^) ν 3409, 3394, 3270, 2919, 2850, 1600, 1550, 1315. MS *m/z*: 427.41 [M + H]^+^. Anal. Calcd For C_26_H_38_N_2_OS: C, 73.19; H, 8.98; N, 6.57. Found: C, 73.02; H, 9.14; N, 6.43.

### 3.4. Tribological Properties

The tribological properties of compounds **2a**–**d**, mixed with TIDTM with the concentration of 1 wt%, were carried out using a UMT-3 microtribometer (Bruker, Campbell, CA, USA). The ball-on-disk configurations of the UMT-3 were used for the coefficient of friction (COF) test. In the tests, steel balls (E52100) with 4.45 mm diameter and hardness (HRC) of 62–63 were used to assemble the friction pair. The balls were cleaned with soap to remove contaminants prior to test the COF of the film.

All tests were performed in a laboratory environment where the temperature was controlled to 25 °C by an air-conditioner. Tests were performed under a rotating speed of 50 rev·min^–1^, a duration of 120 min, and a load of 98 N. The COF was automatically recorded by computer as a function of time, and then the mean COF was calculated. An optical microscope was used to determine the wear scar diameter (denoted as WSD) with an accuracy of 0.01 mm. Each experiment was repeated three times, and the averages of the repeat tests were then calculated and reported in this paper.

### 3.5. Antioxidant Behavior

A Q2000 differential scanning calorimeter (DSC, TA Instruments; New Castle, DE, USA) was employed to evaluate the antioxidant behavior of compounds **2a**–**d**. For non-isothermal method, the samples were scanned from 100 to 400 °C at the heating rate of 20 °C·min^–1^ with a constant flow of nitrogen and oxygen at 30 mL·min^–1^ and 20 mL·min^–1^, respectively. For isothermal method, the samples were scanned at 230 °C under the constant flow of oxygen with 20 mL·min^–1^. Oils were prepared by adding compounds **2a**–**d** into TIDTM at a concentration of 1 wt%, respectively.

## 4. Conclusions

In this work, four novel sulfur-containing 4-aminodiphenylaime derivatized dual functional antioxidants with different action mechanism are designed, synthesized, and confirmed by ^1^H-NMR, FTIR, MS, and elemental analysis. The DSC results showed that adding 1 wt% **2a**–**d** will tremendously increase the oxidative resistance capacity of TIDTM. Especially, compound **2c** exhibited the best antioxidant ability with the OIT of 72.5 min at 230 °C, which is almost 28 times the length of TIDTM. It is believed that the dual functional antioxidants could be attributed to the intramolecular synergism. Moreover, compounds **2a**–**d** do not affect the tribological performance of TIDTM. Based on the above results, compound **2c** appears to be very a promising dual functional antioxidant, especially for use in high-temperature lubricant oils. In addition, this work would also inspire the design of dual functional highly oxidation-resistant antioxidants with different antioxidant mechanisms in the future.

## Supplementary Materials

The supplementary materials are available online, ^1^H-NMR of compounds **2a**–**d**.
