# Supplementary material for: Synthesis, Characterization, and Performance Evaluation of Sulfur-Containing Diphenylamines Based on Intramolecular Synergism
Source: Molecules. 2018 Feb 13;23(2):401. doi: 10.3390/molecules23020401 (PMC6017979; doi:10.3390/molecules23020401)
Supplement: Supplementary file 1 [file molecules-23-00401-s001.pdf]

molecules

MDPI

Supplementary Materials

The ^{1}H-NMR spectra of compounds **2a**-**d** were listed below:

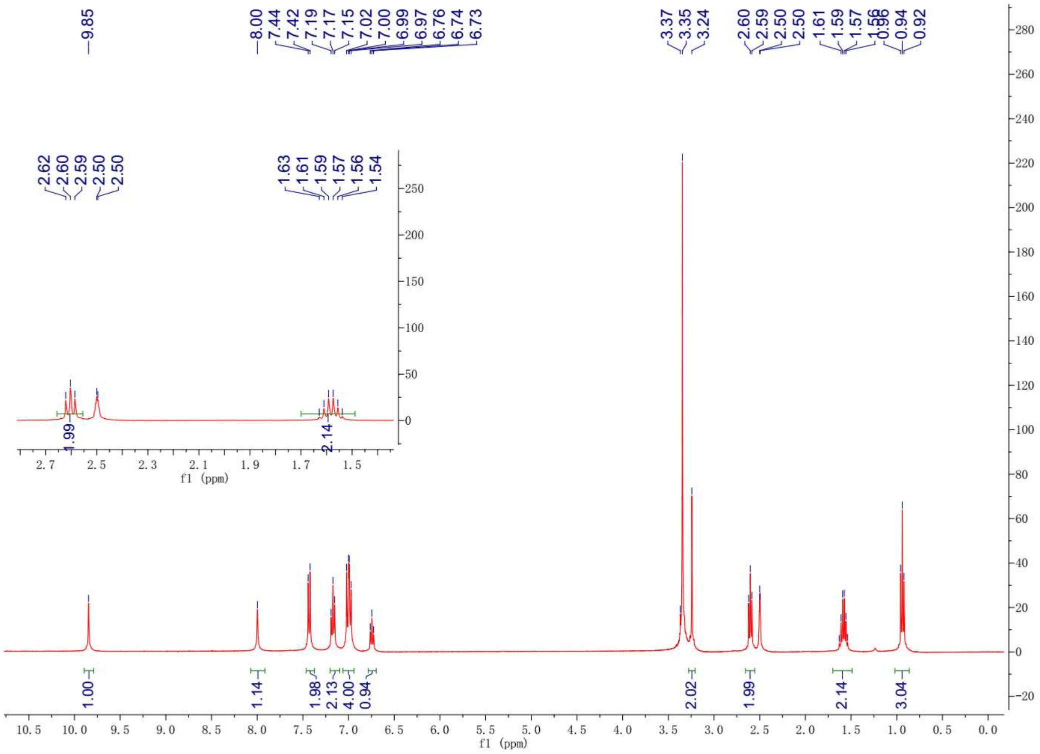

Figure S1. ^{1}H-NMR of compound **2a** (400 MHz, DMSO-d_6)

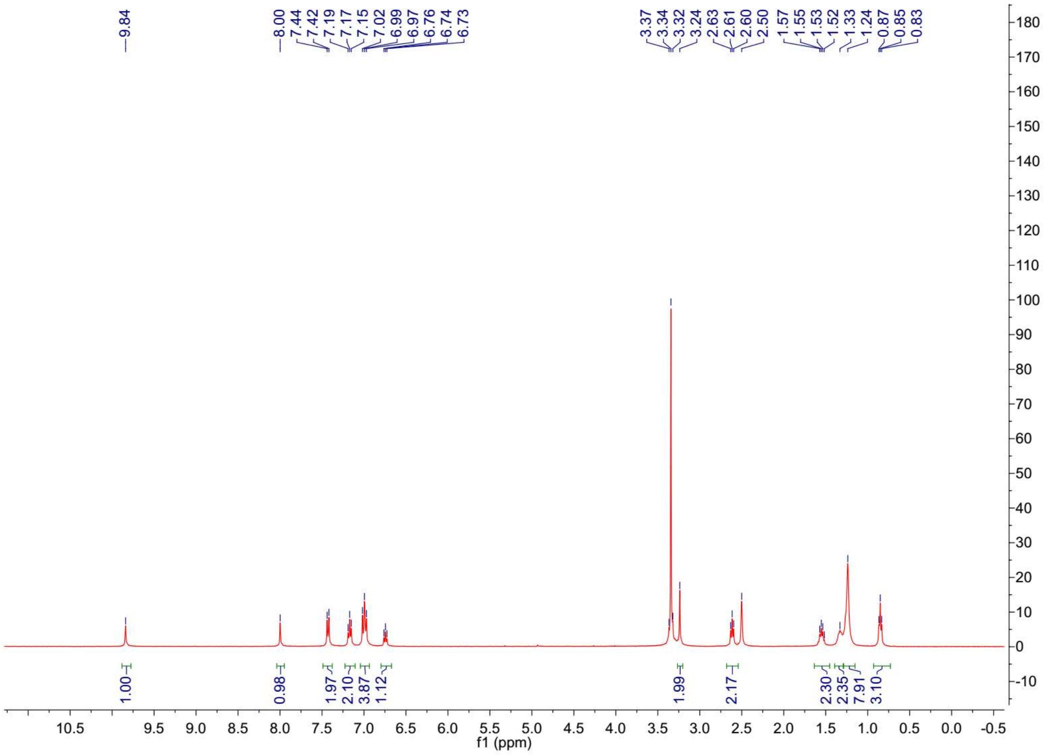

Figure S2. ^{1}H-NMR of compound **2b** (400 MHz, DMSO-d_6)

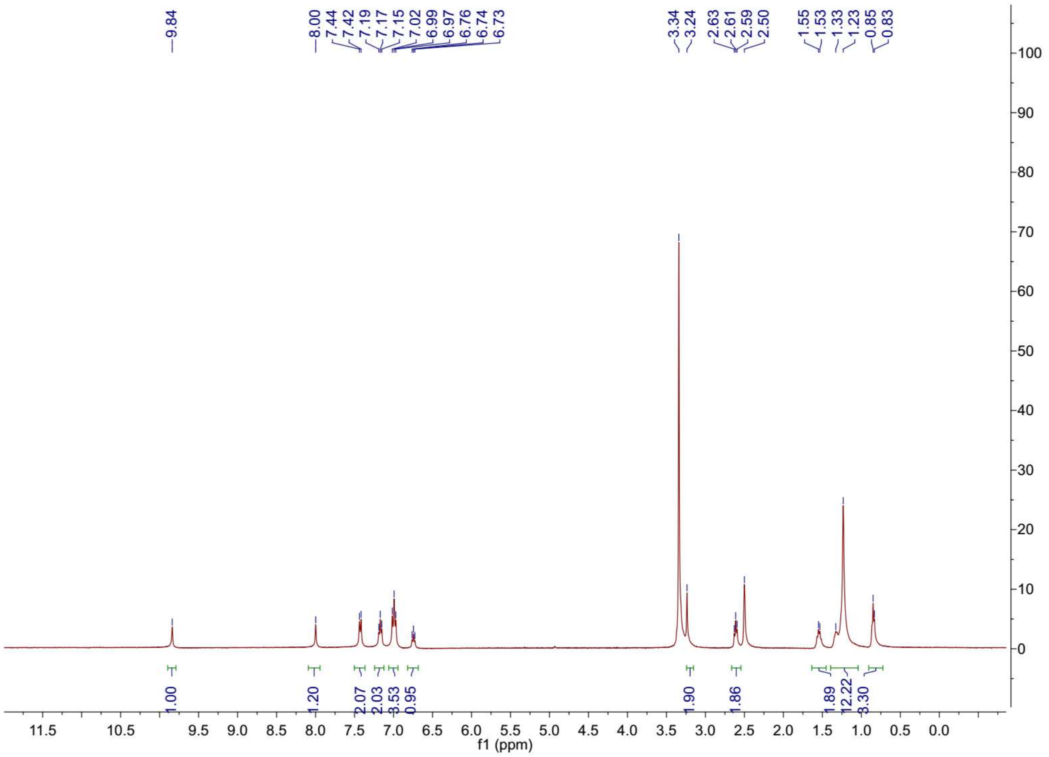

Figure S3. ^{1}H-NMR of compound **2c** (400 MHz, DMSO-d_6)

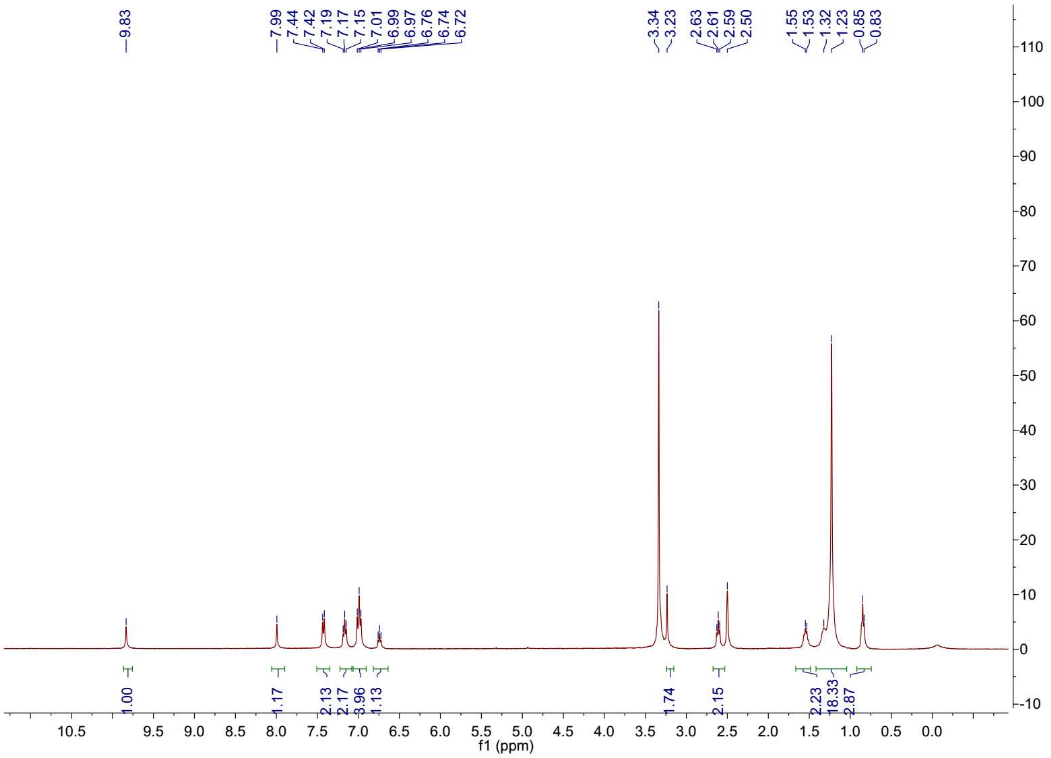

Figure S4. ^{1}H-NMR of compound **2d** (400 MHz, DMSO-d_6)
